# Controversial Roles of Gut Microbiota-Derived Short-Chain Fatty Acids (SCFAs) on Pancreatic β-Cell Growth and Insulin Secretion

**DOI:** 10.3390/ijms21030910

**Published:** 2020-01-30

**Authors:** Jun-Li Liu, Irina Segovia, Xiao-Lin Yuan, Zu-hua Gao

**Affiliations:** 1 The Research Institute of McGill University Health Centre, 1001 Blvd Decarie, Montreal, QC H4A 3J1, Canada; irina.segovia@mail.mcgill.ca (I.S.); zu-hua.gao@mcgill.ca (Z.-h.G.); 2 School of Chinese Medicine, Nanjing University of Chinese Medicine, 138 Xianlin Rd., Nanjing 210023, China; yuanxiaolin@njucm.edu.cn

**Keywords:** microbiota, acetate, propionate, butyrate, histone deacetylase (HDAC), GPR43/FFA2, GPR41/FFA3, glucose-stimulated insulin secretion (GSIS)

## Abstract

In the past 15 years, gut microbiota emerged as a crucial player in health and disease. Enormous progress was made in the analysis of its composition, even in the discovery of novel species. It is time to go beyond mere microbiota-disease associations and, instead, provide more causal analyses. A key mechanism of metabolic regulation by the gut microbiota is through the production of short-chain fatty acids (SCFAs). Acting as supplemental nutrients and specific ligands of two G-protein-coupled receptors (GPCRs), they target the intestines, brain, liver, and adipose tissue, and they regulate appetite, energy expenditure, adiposity, and glucose production. With accumulating but sometimes conflicting research results, key questions emerged. Do SCFAs regulate pancreatic islets directly? What is the effect of β-cell-specific receptor deletions? What are the mechanisms used by SCFAs to regulate β-cell proliferation, survival, and secretion? The receptors FFA2/3 are normally expressed on pancreatic β-cells. Deficiency in FFA2 may have caused glucose intolerance and β-cell deficiency in mice. However, this was contrasted by a double-receptor knockout. Even more controversial are the effects of SCFAs on insulin secretion; there might be no direct effect at all. Unable to draw clear conclusions, this review reveals some of the recent controversies.

## 1. Literature Review

It is estimated that billions of microorganisms live in the human gut, including 1–2 kg of bacteria of ~40,000 different species. They help us absorb ~10% of daily nutrients, protect us from other pathogens, and serve as indicators of abnormalities (e.g., Crohn’s disease, bacterial vaginosis). Bacterial imbalance contributes to obesity, insulin resistance, and bowel and liver diseases. Using non-digestible carbohydrates (e.g., fiber, starch) left in our colon, the gut microbiota generates short-chain (≤6 carbons) fatty acids (SCFAs), which include acetate, propionate, and butyrate. Mimicking “endocrine” signaling molecules, SCFAs were shown to regulate appetite, energy expenditure, and adiposity through central mechanisms, as well as inhibit histone deacetylation, thus permitting gene transcription; most importantly, they were shown to act on G-protein-coupled receptors (GPCRs)—GPR43/FFA2 and GPR41/FFA3—which are widely expressed in many tissues, including pancreatic β-cells. 

Currently, limited results obtained from in vitro, direct stimulations, and in vivo receptor gene targeting are still conflicting, but they seem to suggest that SCFAs are not only nutrient substrates, but also serve as signal molecules capable of improving host metabolism of carbohydrates and lipids. Acting through those two receptors of unique signaling mechanisms (often in a counter regulation), SCFAs seem to directly promote the proliferation, survival, and function of pancreatic β-cells. These actions may contribute to the overall metabolic benefits of maintaining an adequate production of SCFAs and a diversified and balanced microbiome in our gut. 

## 2. Cross-Talks between Microbiota and Their Host through the Production of Mucus, Antimicrobial Signals, and SCFAs 

Defining the human microbiota: A tremendous number of microorganisms, including bacteria, fungi, archaea, and viruses inhabit the human body. From a bacteria-centric view, the gastrointestinal (GI) tract is dominated by two phyla: Firmicutes and Bacteroidetes. Their ratio varies significantly among individuals and is influenced by our health and diseased states. For instance, depletion of a simple species named *Faecalibacterium prausnitzii*, a member of the Firmicutes, is associated with inflammatory bowel disease (IBD) [1]. There is no doubt that we progressed considerably in the analysis of microbiota composition, their key metabolites, and even in the discovery of novel species. However, the most recently published reports simply associate the differences in microbiome under different physiological conditions. More work is needed to go beyond these mere associations and provide direct causal analyses. 

What human microbiota do: Having co-evolved with humans for thousands of years, the gut microbiota makes a significant contribution to human biology and development. It helps process food and digest complex, non-digestible fibers and starch, which generates SCFAs and contributes to our daily energy intake. It also supports colonocyte nutrition and health, promotes adiposity, stimulates lymphoid tissue maturation, and supports our gut structure, morphology, and barrier integrity. Most of these actions can be attributed to the production of SCFAs. In fact, it was shown that germ-free mice gained less weight than their conventionally colonized counterparts, despite having higher caloric intake. They became obese or lean on receipt of fecal microbiota transferred from obese or lean mice, respectively, demonstrating a clear causal link between gut microbiota and the emergence of rodent or even human obesity [2].

How do human microbiota communicate with their host: The composition of the gut microbiota is influenced by health, diet/fat, medication, and the environment. Considering the ability to influence the function of distal organs and systems, the microbiota resembles an *endocrine organ*. SCFAs are major products of bacterial fermentation from insoluble fiber and proteins, and they are signature messengers or “hormones” of the microbiota. For instance, γ-aminobutyric acid (GABA), a key inhibitory neurotransmitter, is produced by *Lactobacillus*. Monoamines, such as noradrenaline, dopamine, and serotonin, are also produced by bacteria. Moreover, the gut microbiota regulates the availability of tryptophan in our circulation, which is critical for the synthesis of 5-hydroxytryptamine (5-HT; serotonin). Furthermore, the gut microbiota is also important for the development of an appropriate stress response, as stress and the activation of the hypothalamic-pituitary-adrenal (HPA) axis influence bacterial composition. 

SCFA levels are closely associated with obesity and diabetes: The gut microbiota clearly influences obesity and the development of type 1 and type 2 diabetes mellitus (T1D and T2D) [3]. In obese mice and humans, there is an increase in Firmicutes (Gram+, producer of butyrate) and a decrease in Bacteroidetes (Gram−, producer of acetate and propionate) [1,4,5]. Indeed, it was reported repeatedly that butyrate levels increase while acetate and propionate levels decrease in obesity [6,7,8,9,10]. Interestingly, the opposite occurs in T2D, i.e., butyrate level decreases and other SCFAs increase. A deficiency in overall SCFA production is also associated with T2D and other diseases [11]. In children progressing to T1D, there is a decrease in bacterial diversity, especially in those producing butyrate and lactate [12]. Conversely, SCFA supplementation is associated with beneficial effects in T2D, for example, butyrate provided a positive effect in both human and animals [13]. A six-month oral administration of acetate (5.2 mg/kg, bid) to diabetic Otsuka Long-Evans Tokushima Fatty (OLETF) rats was well tolerated, decreased weight (fat) gain and lipogenic gene expression, and improved glucose tolerance [14]. In another experiment, an oral administration of acetate and propionate reduced glycemia in diabetic KK-A(y) mice [5]. In all, SCFA supplement seems to improve metabolic health.

In addition to the production of SCFAs, under healthy conditions, the gut microbiota is kept away from its host’s intestinal epithelium by the maintenance of a thick layer of mucus and the production of antimicrobial signals or peptides (such as Reg proteins) [15]. In fact, metabolic disorders are often associated with decreased mucus thickness and antimicrobial defense [16]. Together with the production of SCFAs, these three factors mediate functional cross-talks between microbiota and their host.

## 3. SCFAs Serve as Energy Substrates, Especially for Colonocytes 

Acetate, propionate, and butyrate—the most abundant SCFAs in our system—are produced by the gut microbiota in roughly a ratio of 3:1:1 [17]. Most are directly metabolized in colonocytes; the rest are absorbed into the hepatic portal circulation, providing energy to liver, muscle, kidney, brain, and heart [18]. Absorbed into gut epithelia, acetate enters the tricarboxylic acid (TCA) cycle (also known as the citric acid cycle or the Krebs cycle) after being converted to acetyl CoA, and it produces ATP, cholesterol, and fatty acids [19]. Propionate is a substrate for gluconeogenesis in the intestine before reaching the liver. Elevated de novo synthesized glucose from the gut epithelium is sensed by a gut-brain neural circuit originating from the portal vein, which increases insulin sensitivity and glucose tolerance [19,20,21]. In the colonocytes, most butyrate undergoes β-oxidation in the mitochondria to generate acetyl CoA, which then enters the TCA cycle and generates energy in the form of ATP [22]. Through radio-labeled tracing starting from human colon, ~36% acetate, 9% propionate, and only 2% butyrate were found unaltered in the general circulation [19]. Their normal blood concentrations in human and rodents range from 200 µM acetate and 1 to 20 µM propionate and butyrate [18,23]. Furthermore, different SCFAs can be interconverted by the gut microbiota. For example, 24% acetate becomes butyrate and, conversely, 10% butyrate can be converted to acetate [19]. 

Several studies advanced the energy harvesting hypothesis, whereby the generation of SCFAs contributes additional calories through fiber fermentation. However, it was also observed that, in humans, high-fiber diets protect against weight gain by reducing inflammation, by sensitizing tissues to insulin, and by elevating satiety [24]. In the liver, acetate contributes to de novo lipogenesis while propionate reduces adipose content, demonstrating rather unique roles of each individual SCFA [24]. Thus, it would be an over simplification to consider the overall level of SCFAs as either good or bad for host metabolic health. There are significant changes in fecal bacterial compositions of T2D vs. healthy individuals [25], with a ~30% decrease in fecal contents of propionate and butyrate in T2D subjects [26]. On the other hand, higher fecal concentration of SCFAs is associated with metabolic risk factors including adiposity, waist circumference, and homeostatic model assessment (HOMA) index [27]. This association does not mean that SCFAs are “detrimental,” because SCFAs are not just energy substrates like palmitate, a long-chain saturated fatty acid [28,29,30]. In fact, SCFAs also regulate metabolic, immune, and intestinal functions as signaling molecules, which is widely reported. 

### SCFAs Improve Host Metabolism by Regulating Appetite and Energy Expenditure

Increased dietary fiber promotes weight loss and improves glycemic control through SCFA production. The impact of an SCFA-enriched diet provides a direct, causal link. It was reported that mice fed a butyrate-enriched high-fat diet (HFD) have increased energy expenditure and are resistant to obesity [31]. Furthermore, butyrate increased the production of GLP-1, peptide YY (PYY), and growth hormone; it also reduced appetite, activated brown adipose tissue, and diminished diet-induced obesity [21,32]. It was also proven in humans that the administration of inulin-propionate ester (which produces SCFAs) significantly increases postprandial GLP-1 and PYY levels while reducing caloric intake and fat mass [18,20]. Furthermore, oral gavage of acetate reduces weight gain and improves glucose tolerance. Both acetate and propionate reduce appetite via central mechanisms [18,33]. SCFAs seem to positively affect glucose metabolism by normalizing plasma glucose levels and glucose tolerance, and they seem to promote fatty acid oxidation, decreasing fat storage and, thus, preventing obesity [5]. On the other hand, in rodents fed an HFD, increased acetate production from gut bacteria stimulates a central parasympathetic mechanism, which leads to ghrelin secretion, hyperphagia, and increased glucose-stimulated insulin secretion (GSIS), promoting caloric storage and adiposity [34,35]. To be noted, that conclusion was based heavily on the uses of vagotomy and vagal blocker atropine, which alone have profound metabolic consequences. The seemingly conflicting result presents a *detrimental* influence of acetate on host metabolism, although it may still be consistent with our notion that SCFAs stimulate β-cells directly (see below). 

## 4. SCFAs Interact with G-Protein-Coupled, Nutrient-Sensing Receptors and Histone Deacetylases (HDACs)

**Figure 1:**
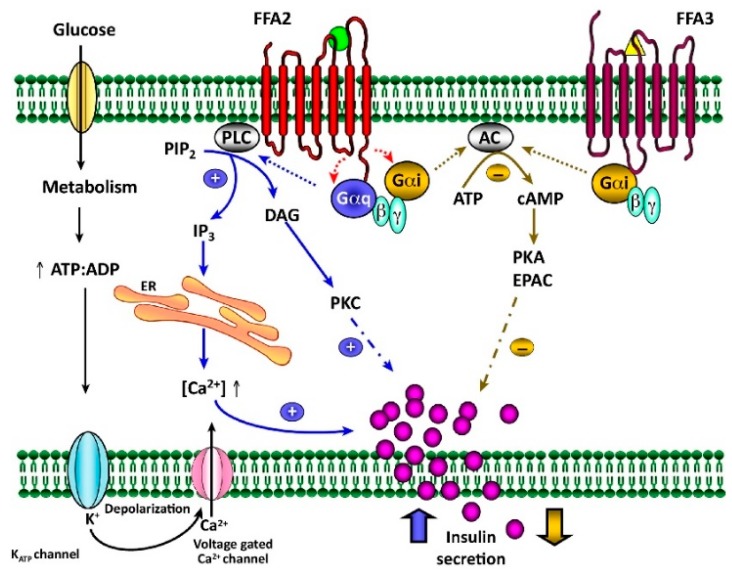
Regulation of insulin secretion by short-chain fatty acids (SCFAs) through receptors FFA2 and FFA3. SCFAs can bind to both receptors either amplifying (in blue) or diminishing (in golden) glucose-stimulated insulin secretion (GSIS). Upon ligand activation of FFA2, Gαq/11 subunits activate PLC, which hydrolyzes PIP_2_ to DAG and IP_3_. In turn, DAG activates protein kinase C (PKC) and IP_3_ releases Ca^2+^ from ER stores, both amplifying the insulin release. FFA2, like FFA3, can also couple with Gαi/o subunits and inhibit AC, which decreases cAMP level, inhibiting PKA and EPAC-mediated insulin release [18,37]. Adopted with permission from Trends Endocrinol Metab (License No. 4724910996230).

In 2003, GPCRs GPR41 and GPR43 were deorphanized and renamed FFA3 and FFA2, respectively; SCFAs were established as their cognate ligands, which firmly established them as signaling molecules [18]. Upon ligand binding, FFA2 (GRP43/FFAR2) activates either pertussis toxin (PTX)-sensitive Gαi/o or PTX-insensitive Gαq/11 proteins, causing changes in intracellular cAMP or calcium/protein kinase C (PKC), respectively (Figure 1). As the actions of these two pathways often contradict each other, we speculate that there could be two separate populations of β-cells, as evidenced by the exclusive presence of Gαq/11 in insulinoma MIN6 cells and Gαi/o in INS1 cells [36], although these are transformed β-cells from different species of mouse and rat, respectively. Acetate and propionate are the most potent activators of receptor FFA2 with an EC_50_ of ~20 to 300 µM. For propionate, the latter concentration would be considered supra-physiological given its peak serum level of less than 20 µM [20]. 

With only 33% sequence identity to FFA2, FFA3 (GRP41/FFAR3) couples exclusively to Gαi/o and mediates a decrease in cellular cAMP level. The two receptors differ in affinity for different SCFAs, in tissue distribution, and perhaps in physiological functions [5]. Ligand affinity to FFA3 is in the following order: propionate (EC_50_ ≈12 µM) > butyrate >> acetate [20]. Both receptors are widely expressed in major tissues, including islet α- and β-cells [23,38]. The ligand affinities and specific agonists or antagonists are currently being developed, as listed in Table 1 [36,39]. In addition, studies indicated that SCFA binding to FFA2 also recruits β-arrestins, presumably leading to receptor internalization and G-protein–independent signaling; this is not known to occur for FFA3 [40]. In human monocytes, FFA2 and FFA3 were shown to form a heterodimer with markedly enhanced recruitment of β-arrestins [41]. Indeed, the heterodimer displayed distinct signaling preference from either of the parental homomers, e.g., more p38 but less cAMP regulation [41].

Histone (de)acetylation is a central switch that allows interconversion between permissive (acetylation) and repressive structures (deacetylation) of the chromatin. Acetyl groups are added to histone tails by histone acetyltransferases (HATs) and removed by deacetylases (HDACs). Perhaps independent of the interaction with membrane receptors, SCFAs can be brought into the cells through transporter sodium-coupled monocarboxylate transporter 1 (SMCT-1), occupy the active site of HDACs, and cause an inhibition [48]. Butyrate, propionate, and acetate are all HDAC inhibitors although with decreasing potencies. HDAC inhibition in general promotes chromatin acetylation and target gene transcription, thus potentially influencing cellular function, although it is unclear how cell and gene *selectivity* can be achieved. Beyond metabolism, SCFAs also protect the integrity of the gut epithelium by limiting the growth of pathogenic bacteria. They are considered as therapeutic modalities against intestinal disorders and leaky gut-derived metabolic endotoxemia associated with obesity, inflammation, and insulin resistance [18]. With these benefits in sight, exploring the roles of SCFAs in coordinating various biological processes is an exciting area of investigation. Current evidence indicates that all three forms of SCFAs have varying efficiencies, or sometimes even different effects. Their sites of action vary greatly from the gut, liver, fat tissue, to the brain. 

## 5. Conflicting Results of SCFA Receptor Knockouts on Glucose Tolerance, Insulin Secretion, and β-Cell Mass 

FFA2 gene deficiency caused impaired glucose tolerance and insulin secretion after prolonged HFD feeding [36]. Receptor FFA2 is linked to two G-proteins leading to opposing effects; Gαq/11-mediated signaling was observed to augment β-cell expansion, whereas Gαi/o-mediated signaling was observed to restrict it [18]. Measured in mice at the messenger RNA (mRNA) level, FFA2 is widely expressed in the GI tract, adipose tissues, and the pancreas. It is also widely expressed in most β-cells, as determined by immunohistochemistry (IHC). Its level was significantly induced upon HFD-induced obesity [36]. In humans, FFA2 is also colocalized with insulin in β-cells, and the protein level can be detected by Western blots [49]. Under a chow diet, or following a short-term (eight weeks) HFD, FFA2 knockout (KO) mice (Lexicon) exhibited no change in body weight, food intake, relative tissue weights, insulin secretion, and glucose tolerance. A prolonged HFD to 14 weeks, however, caused glucose intolerance and hyperglycemia due to impaired insulin secretion in response to either glucose or arginine. Significant β-cell expansion and proliferation were induced by HFD in wild-type mice, which was largely abrogated by FFA2 KO. There was also a decreased islet density, insulin content, and expression of insulin and key β-cell markers (MafA, Pdx1, NeuroD) in FFA2 KO mice [36]. In ex vivo experiments, FFA2 agonist phenylacetamide (PA, compound 58) potentiated glucose- and arginine-stimulated insulin secretion in wild-type islets. Consistent with these findings, PA or acetate enhanced cell proliferation, insulin level, and Pdx1 and NeuroD gene expression in MIN6 cells, which was mediated via an association with Gαq/11. These results suggest that FFA2 is normally expressed in β-cells, and that its activity is essential for β-cell compensation in response to obesity in mass and insulin secretion through Gαq/11- and PLC-mediated IP_3_/Ca^2+^ pathways; thus, it is a potential therapeutic target [36]. 

Supporting the above report, another FFA2 KO also caused a reduction in β-cell mass due to increased β-cell death. Gαq-biased FFA2 agonists 2-butynoic acid (SCA15) and 2-propynoid acid (SCA14) stimulated islet cell proliferation [50]. Similarly, female FFA2 KO mice showed reduced β-cell mass and cell proliferation during pregnancy in another report [51]. Increased SCFAs were found in the cecal lumen of pregnant mice, together with elevated FFA2 expression in the islets, both of which may stimulate β-cell compensation against insulin resistance. Based on these reports, activation of FFA2 receptor by SCFAs would be stimulatory to β-cell proliferation and insulin secretion. Nevertheless, prior to this, there was an earlier, conflicting report. After a 55-bp deletion of the coding region in exon 3 of the FFA2 gene and upon HFD, FFA2 KO mice exhibited lower fat mass and improved glucose tolerance and insulin sensitivity, although possible changes in β-cell mass and insulin secretion were not measured [52]. 

A combined FFA2 and FFA3 gene deficiency *improved* glucose tolerance and insulin secretion [53]. FFA3 mRNA expression was enriched in mouse pancreatic islets, which was significantly higher than in the duodenum and other tissues, although the detection in protein level was challenged by a lack of a suitable antibody [54]. Using a fluorescent reporter gene (mRFP), directed under either FFA2 or FFA3 promoter, and co-stained with insulin antibody, it became clear that most β-cells express both receptors FFA2 and FFA3 [53]. 

In cultured MIN6 and human βH1 cells, addition of 100 nM GLP-1 on 11 mM glucose significantly stimulated insulin release. Pretreatment of acetate at a physiological range of 0.1 to 1 mM caused a dose-dependent decrease in GLP-1-stimulated insulin release. This effect was abolished by a pretreatment of PTX, which inhibits Gαi activity, known to be associated with the signaling of both FFA2 and FFA3 [53]. Indeed, small interfering RNA (siRNA)-mediated receptor knockdown further demonstrated that the inhibition by acetate was dependent on the normal expression of both FFA2 and FFA3, whereas individual knockdowns did not affect it. The acetate inhibition on GLP-1-stimulated insulin secretion was further confirmed using isolated mouse islets, which was also sensitive to PTX pretreatment. Finally, using FFA2 and FFA3 knockout mice, it was shown that one needs to knockout both FFA2 and FFA3 in order to abolish acetate inhibition [53], suggesting that FFA2/3 together mediate a *net inhibitory* influence, in conflict with previous FFA2 KO results [36].

In this report, there was no morphological change in the islets of FFA2 or FFA3 single knockouts in chow diet, and there was no change in β-cell mass either [53]. Loss of both FFA2 and FFA3 receptors, however, *improved* insulin secretion and glucose tolerance under HFD, but had *no effect* on β-cell mass. These subtle changes, or lack of, suggest instead that the normal expression of both receptors enabled a *net negative* influence on β-cell function. Finally, a combinational, β-cell-specific targeting of both receptors in adult β-cells was established using a mutant mouse of FFA2^lox/lox^, FFA3^−/−^. In it, FFA3 was permanently deleted, and FFA2 deletion was inducible by RIP-Cre expression. Once again, double deficiencies of both receptors resulted in improved glucose tolerance [53]. In sum, FFA2/3 double KO improved glucose tolerance and insulin secretion but caused no change in β-cell mass; single KOs had no effect (done mostly on chow diet), and acetate inhibited insulin secretion via FFA2/3 receptors and their interaction with Gαi. It was proposed that normally FFA2 and FFA3 mediate a net inhibition on insulin secretion from β-cells (see Section 7 for more), which does not affect glucose tolerance. This inhibition would become relevant or even critical in diabetes because of increased acetate formation within the β-cells, as reported in Reference [53].

This report was partially supported by a follow-up report of FFA3 knockout, which improved glucose tolerance, insulin sensitivity, and insulin secretion with a reduced β-cell mass [54], but was largely in contrast with McNelis et al.’s findings (see Section 5) [36]. Chief among the differences from FFA2 and dual receptor knockouts are whether FFA2 KO alone has an effect, whether FFA2 or dual receptor KO impaired or improved glucose tolerance, whether insulin secretion was decreased or increased, and whether β-cell mass was decreased or unchanged. Moreover, why does FFA2 agonist, PA, stimulate insulin secretion, while acetate inhibits it (see Section 7)? In reflection, we suspect that Tang et al. [53] did not detect significant changes in individual KOs at least in part due to strain-specific or technical variabilities. When the two receptors were ablated together, the positive outcome in insulin secretion and glucose tolerance may suggest that, normally, FFA3 has a negative but dominant effect over FFA2; the latter seems to be more pro-islets. 

## 6. Acetate and Propionate Protect β-Cells from Cytokine-Induced Damage 

Current evidence suggests a protective role for SCFAs on β-cells, and endogenous FFA2 expression seems to be essential for this. Its knockout caused increased β-cell death (loss) in mice at three and 10 weeks of age (see also Section 5) [36,50]. In fact, there was a 2–4-fold increase in the serum level of unmethylated preproinsulin DNA—a biomarker of β-cell death measured by fluorescent-based multiplex PCR [55], and a 1.6-fold increase in caspase-3 cleavage in islet cells measured by quantitative IHC [50]. 

More directly in cultured human islets, 24-h pretreatment with a high 1 mM propionate significantly reduced cytokine- and palmitate-induced cell death [49]. Moreover, in human islets, 20-h treatment with cytokines caused significant apoptosis and a 3.5-fold increased caspase-3/7 activities. Pretreatment with a high 1 mM sodium acetate largely rescued cell death. Similarly, in mouse islets, 20-h cytokine and palmitate treatment caused apoptosis, which was rescued by either 1 mM sodium acetate or propionate, both of which were very high doses [56]. This direct and indirect evidence clearly demonstrated that SCFAs protect β-cells from the damages caused by free fatty acids (FFAs) and cytokines, and suggested a role of FFA2 in signaling. To be noted, the effect of butyrate was not tested. Based on these reports, we can conclude that both sodium acetate and propionate protected cytokine- or palmitate-induced islet cell death; FFA2 deficiency caused β-cell death and reduced β-cell mass, while SCFAs acted through FFA2-protected islet β-cells. Nevertheless, as minor but conflicting evidence in MIN6 cells, pretreatment with FFA2 agonist (PA) and acetate had no effect on palmitate-induced caspase-3 activation and apoptosis [36]. 

## 7. Conflicting Evidence That SCFAs Regulate Insulin Secretion from Pancreatic β-Cells 

It is generally accepted that a healthy diet—rich in fiber, fruit, and vegetable—generates more SCFAs, which benefit host metabolism [48]. Being central to metabolic regulation by producing insulin and glucagon, pancreatic islets are expected to benefit from increased SCFAs in maintaining cell mass and function, either directly or indirectly, as a result of increased GLP-1 release or improved metabolic condition. This seems to be supported by most receptor knockout reports discussed above. However, those studies did not focus on a direct effect on the islets. Insulin secretion has been studied by static incubations of isolated islets, ex vivo dynamic perfusion of the islets, or in situ whole pancreas perfusion. Dated ~30 years ago, there were reports that SCFAs regulate insulin secretion, although a clear consensus was not achieved [38]. For instance, in 1987, in islet tumor lines, incubation with sodium butyrate (2 mM, which is a very high concentration) for 1–3 days changed cell morphology and inhibited cell proliferation, but increased insulin and glucagon mRNA levels [57]; in 1978, using sheep islets, very high doses of 2.5–10 mM butyrate (more than acetate and propionate) stimulated insulin secretion [58]. Both indicated that butyrate stimulates insulin production or secretion; this was, however, based on the use of a very high concentration, which would never be seen in normal circulation. 

### 7.1. FFA2 Agonist (PA) Potentiates Insulin Secretion through Gαq/PLC-Mediated IP_3_ and Ca^2+^ Activations 

Using static incubated mouse and human islets, as well as MIN6 cells, FFA2 agonist PA (compound 58) was shown to stimulate GSIS [36]. The effect in mouse islets was significantly abolished after FFA2 knockout. Furthermore, siRNA-mediated knockdown of either FFA2 or its associated Gαq/11 in MIN6 cells blocked the stimulation of FFA2 agonist on insulin secretion [36]. PA also stimulated IP_3_ production in mouse and human islets; it boosted Ca^2+^ current, but had no effect on cellular cAMP level in MIN6 cells. This report provided clear evidence that PA or other SCFAs, acting through FFA2-associated Gαq/11, PLC, IP_3_, and Ca^2+^, potentiates GSIS. 

### 7.2. Propionate and Acetate Potentiate GSIS in Human and Mouse Islets

In 2017, addition of 10 to 1,000 µM propionate to perifused human islets (measuring dynamic insulin secretion) induced an immediate and significant further elevation in GSIS [49]. In a follow-up report, this propionate-induced potentiation was abolished in islets isolated from FFA2 KO mice, suggesting the involvement of the receptor [55]. Furthermore, since FFA2 is linked to three possible pathways influencing GSIS—(1) Gαi and cAMP production; (2) Gαq/PLC, IP_3_ and Ca^2+^; (3) Gαq/PLC, DAG, and PKC—their contributions in propionate’s effect was explored. Indeed, propionate potentiation on insulin release was completely abolished by phorbol ester and PKC inhibitor 4β-phorbol 12-myristate 13-acetate (PMA), but was unaffected by 4α-phorbol 12,13-didecanoate (PDD), an inactive phorbol ester, indicating a PKC-mediated effect. In addition, propionate caused a significant increase in calcium current (suggesting an IP_3_-triggered effect) [49], further indicating the involvement of both Gαq/PLC-associated DAG/PKC and IP_3_/Ca^2+^ pathways.

Similarly, the addition of acetate (1 to 1,000 µM) also boosted GSIS rapidly in perifused mouse islets, and this effect was abolished in FFA2 KO islets [55]. The potentiation on insulin secretion was further abrogated by the pretreatment of PLC inhibitor—which blocks both Ca^2+^ and PKC pathways—and by PMA, which depletes PKC. The addition of acetate to high-glucose-treated cells triggered a further increase in intracellular Ca^2+^ levels, determined by microfluorimetry; this effect was also dependent on FFA2. On the other hand, pretreatment with PTX, which abolishes the Gαi effect through ADP-ribosylation, had no effect on acetate-induced potentiation [55]. Thus, the acetate effect seems to be mediated by FFA2-Gαq and consequent activations of both IP_3_/Ca^2+^ and DAG/PKC pathways, rather than by a reduction in cellular cAMP level. To be noted, the effect of butyrate was not tested using a similar model of dynamic insulin release. 

### 7.3. Contradictory Receptor- and G-Protein-Dependent Effects of Acetate and Propionate on GSIS

In contrast to the above reports described in Section 7, it was recently reported that acetate *inhibits* GLP-1-induced insulin secretion via both FFA2 and FFA3 receptors (see Section 5) [53]. This seems to support a dominant and negative effect of FFA3 over FFA2. This is consistent with a much earlier observation in 1981, where 1 mM acetate reduced arginine-induced insulin secretion in perfused rat pancreas [59], but contradicts two other observations: one in 1979, where 1 mM acetate potentiated GSIS in rat islets [60], and another one in 1977, where acetate infusion (0.4 µM/min) in rats significantly potentiated GSIS [61]. Supporting an inhibitory role of FFA3, islets of FFA3 KO mice (Masashi Yanagisawa, Texas) exhibited elevated insulin secretion with no change in insulin content [47]. Propionate, but not butyrate, also *inhibited* GSIS from wild-type islets, which was abolished by FFA3 KO. Similar inhibition was confirmed using MCPC (an FFA3 agonist) which showed sensitivity to PTX, thus indicating the involvement of Gαi [47]. 

On the other hand, FFA2 KO mice demonstrated decreased insulin secretion and glucose infusion rate (indicating decreased insulin secretion and/or glucose disposal) during hyperglycemic clamp [38]. Acetate at 1 mM high concentration *potentiated* GSIS from ex vivo cultured wild-type mouse islets but did not affect GSIS from FFA2 KO islets. Even more interestingly (and confusingly), selective FFA2 agonists produced either positive or negative effects on GSIS, perhaps due to their preferential links with either Gαq or Gαi pathways [38]. Thus, incubation with FFA2 agonists SCA14 and SCA15 potentiated GSIS (like that of high-dose acetate), which was dependent on Gαq-mediated PLC activation. However, CMTB, another FFA2 agonist, caused a decrease in GSIS by activating the PTX-sensitive Gαi pathway. Based on these two recent reports [38,53], acetate acting on either FFA2 and FFA3, which can couple to either Gαi and Gαq under different conditions, may exhibit very different effects on GSIS. 

From several KO reports, there seemed to be dimorphic effects when FFA2 was coupled to either Gαi or Gαq protein, and there also seemed to be different responses in rodent vs. human islets, in addition to the differences mediated by either FFA2 or FFA3. For a given agonist of FFA2, the effects may vary depending on cellular environment, including perhaps cell-specific G-protein coupling. For instance, in MIN6 cells, PA only acted through FFA2/Gαq/11 then IP_3_ production, and it stimulated insulin secretion, whereas, in INS-1 cells, PA only acted through (mediated by FFA2, FFA3, or both) Gαi then cAMP reduction, and it inhibited insulin secretion [36]. More specific and in-depth studies may help, for example, to elucidate the role of FFA2 in *human islets,* especially on GSIS. To be noted, the physiological effect of butyrate on GSIS, if any, was not reconfirmed in more recent studies using more accurate or dynamic assays.

Finally, there might be no direct effect of SCFAs on β-cells at all. Using in situ perfused whole pancreas in mice, none of the SCFAs at physiological concentrations exhibited any significant and direct effect on insulin secretion. Only butyrate (at a very high concentration of 10 mM) and FFA2 agonist CFMB seemed to inhibit GSIS through a proven stimulation on somatostatin release. The latter is known to inhibit insulin secretion. Hence, at least some of the positive or negative effects on host metabolism are likely caused by indirect actions of SCFAs on other tissues, cells, or processes [45].

### 7.4. Our Interpretation

Based on earlier and more recent observations made using in vivo, in vitro, and ex vivo approaches, we can safely propose that FFA2 agonists, especially when clearly acting through Gαq/11, would stimulate insulin secretion, while FFA3 agonists acting through Gαi/0 would only cause an inhibition. When both receptors are co-expressed, the negative effect of FFA3 seems to dominant over the positive influence by FFA2.

No effect of SCFAs was found on α-cells and glucagon secretion. Unlike the reports on β-cells, there was no study of pancreatic α-cell proliferation or function, although both receptors—FFA2 and FFA3—are expressed on α-cells [23]. There was no sign of any α-cell defect in FFA2^−/−^ mice either [36], and neither was there evidence that SCFAs regulate α-cell secretion. Increasing concentrations of acetate (0.1 to 1 mM), under both 2.8 and 16.7 mM glucose, had no effect on glucagon secretion either [53].

Do SCFAs promote islet function by inducing Reg family genes? In islet β-cells, the gut microbiota, through the release of SCFAs into circulation, promotes the production of cathelicidin-related antimicrobial peptides (CAMP, CRAMP), which not only protect the islets against autoimmune diabetes (in NOD mice and BBdp rats), but also promote β-cell proliferation, survival, and insulin secretion [62,63,64]. Cathelicidins represent a family of host defense peptides including Reg proteins, which we studied for over a decade and for which we established their roles in β-cell survival, expansion, and function [65,66,67,68,69,70]. Elsewhere in the gut, microbiota and the local production of SCFAs induce gastric and intestinal expression of Reg-3β and -3γ genes in goblet and columnar cells [71,72,73]. Reg proteins are normally produced in pancreatic acinar and islet cells in higher quantities than in the intestines. Since cathelicidins and Reg proteins share a high degree of structure similarity, we believe that Reg proteins may also be induced by SCFAs from the pancreas, which would mediate at least part of the effects on endocrine islets.

## 8. Summary

Both receptors FFA2 and FFA3 are normally expressed in pancreatic β-cells (FFA2 levels can be induced by obesity). Activation of these G-protein-coupled receptors results in changes in cAMP and Ca^2+^ levels and PKC activities. It is, therefore, feasible for FFA2 agonists to stimulate β-cell growth and insulin secretion through Gαq/PLC and consequent activations of either the IP_3_/Ca^2+^ or DAG/PKC pathway. However, the results of different receptor knockouts are still contradictory on the change in β-cell mass. Even using in vitro, direct measurement, the effects of different SCFAs on GSIS are contradictory. Will additional and longer *pretreatment* of SCFAs boost insulin secretion even further? Will SCFAs alone without high glucose affect *basal* insulin synthesis or secretion? We take for granted that SCFAs regulate β-cells exclusively via FFA2, FFA3, or both, and that acetate, propionate and butyrate share the same receptors. These notions may not be absolutely true (e.g., acetate is much more polarized and water-soluble than butyrate). A recent microbiome-wide association study showed that increased production of butyrate was associated with improved insulin response of the β-cells, whereas increased fecal propionate level was causally related to the incidence of T2D [74]. Thus, different SCFAs may indeed play quite opposite roles. Another question that arises is whether various SCFAs can be transported into cells in a different fashion or rate, thus bypassing the membrane receptors. Finally, the effects of butyrate and HDACs remain to be examined.

## Figures and Tables

**Table 1:** Properties of short-chain fatty acids (SCFAs), their receptors, and associated ligands [40,42,43]. Compounds (1) and (2) were found to activate FFA2, then either Gαi, Gαq, or β-arrestin-2 [44]. Otherwise, there are only very limited reports in patent literature (https://books.google.com/advanced_patent_search), e.g., US20080312277A1, WO2003057730A1. Further studies on orthosteric binding capacity, high-affinity ligand, and potency are essential to unravel therapeutic potential of targeting these receptors.

	GPR41/FFA3	GPR43/FFA2
	Ligand affinity (EC_50_, µM)	
Acetate	>1000	35 to 431
Propionate	6 to 127	14 to 290
Butyrate	42 to 158	28 to 371
		
Ligand preference	Propionate > Butyrate > Acetate	Acetate = Propionate > Butyrate
Coupled G-proteins	Gαi/o	Gαq/11, Gαi/o, β arrestin
Agonists	MCPC or MCP, C1 to C6, Compound 4 [45]	CMTB, phenylacetamide (PA; Comp 58), CFMB [45], SCA14/15, C1 to C5, A [46]
Orthosteric agonist	AR19 [44]	Compound (1) and (2) [44] Compound (3) [44]
Orthosteric antagonist		GLPG0974 = (4) [44] CATPB = (5) [44]
Allosteric agonist	(12), AR420626 = (13) [44]	4-CMTB = (10) [44]
Antagonists	1 [36,39], β-hydroxybutyrate (BHB)? [44,47]	
